# Genomic Surveillance and Resistance Profiling of Multidrug-Resistant *Acinetobacter baumannii* Clinical Isolates: Clonal Diversity and Virulence Insights

**DOI:** 10.3390/microorganisms13112429

**Published:** 2025-10-23

**Authors:** Maria Vittoria Ristori, Ilaria Pirona, Lucia De Florio, Sara Elsa Aita, Gabriele Macari, Silvia Spoto, Raffaele Antonelli Incalzi, Silvia Angeletti

**Affiliations:** 1 Research Unit of Clinical Laboratory Science, Department of Medicine and Surgery, Università Campus Bio-Medico di Roma, Via Alvaro del Portillo, 21, 00128 Rome, Italy; m.ristori@policlinicocampus.it (M.V.R.); l.deflorio@policlinicocampus.it (L.D.F.); 2 Operative Research Unit of Laboratory, Fondazione Policlinico Universitario Campus Bio-Medico, Via Al-varo del Portillo, 200, 00128 Rome, Italy; 3 GenomeUp SRL, 00144 Rome, Italy; ilariap@genomeup.com (I.P.); gabriele@genomeup.com (G.M.); 4 Istituto Di Patologia Speciale Medica, Catholic University of the Sacred Heart, 00168 Rome, Italy; 5 Operative Research Unit of General Surgery, Fondazione Policlinico Universitario Campus Bio-Medico, 00128 Rome, Italy; s.aita@policlinicocampus.it; 6 Diagnostic and Therapeutic Medicine Department, Fondazione Policlinico Universitario Campus Bio-Medico, 00128 Rome, Italy; s.spoto@policlinicocampus.it; 7 Operative Research Unit of Internal Medicine, Fondazione Policlinico Universitario Campus Bio-Medico, Via Alvaro del Portillo, 200, 00128 Roma, Italy; r.antonelli@policlinicocampus.it; 8 Research Unit of Internal Medicine, Department of Medicine and Surgery, Università Campus Bio-Medico di Roma, Via Alvaro del Portillo, 21, 00128 Roma, Italy

**Keywords:** *Acinetobacter baumannii*, multidrug resistant, genomic surveillance, resistance profiling

## Abstract

*Acinetobacter baumannii* is a multidrug-resistant opportunistic pathogen that poses critical challenges in hospital settings due to its environmental resilience and high resistance to antibiotics. Genomic surveillance has become essential for identifying transmission patterns, guiding antimicrobial stewardship, and informing infection control policies. We conducted whole-genome sequencing on 44 *A. baumannii* isolates collected between 2022 and 2023 from diverse wards in an Italian hospital. Illumina-based sequencing was followed by a comprehensive bioinformatics pipeline, including genome assembly, taxonomic validation, MLST, SNP-based phylogeny, pan-genome analysis, antimicrobial resistance (AMR) gene profiling, and virulence factor prediction. Most isolates were classified as ST2; SAMPLE-34 was ST1 and genetically distinct. Phylogenetic analysis revealed four clonal clusters with cluster-specific AMR and accessory gene content. The pan-genome included 5050 genes, with notable variation linked to hospital ward origin. ICU and internal medicine strains carried higher loads of AMR genes, especially against aminoglycosides, β-lactams, and quinolones. Virulence profiling highlighted widespread immune evasion mechanisms; “Acenovactin” was predominant, while some isolates lacked key adhesion or toxin factors. Our findings underscore the clinical relevance of integrating genomic epidemiology into routine hospital surveillance. Identifying clonal clusters and resistance signatures supports real-time outbreak detection, risk stratification, and targeted infection prevention strategies.

## 1. Introduction

*Acinetobacter baumannii*, a Gram-negative pathogen, is one of the most important multidrug-resistant bacteria and has emerged as a primary cause of both hospital- and community-acquired infections [1]. *A. baumannii* is responsible for a wide range of diseases, including bloodstream infections, ventilator-associated pneumonia, urinary tract infections, skin infections, and meningitis [2]. The World Health Organization (WHO) has identified this bacterium as a critical priority pathogen [3], particularly due to specific clones (IC-I, IC-II, and IC-III) that are considered high-risk pandemic clades with significant persistence in hospital settings [4]. Despite substantial global efforts in *A. baumannii* surveillance, localized genomic insights within specific hospital sub-settings remain limited. This gap hinders the capacity to link genomic features with epidemiological contexts and to inform ward-specific infection control strategies. *A. baumannii* has a significant impact on public health, contributing over 7000 infection cases and 500 deaths annually [5]. Its impact is amplified by the limited availability of effective diagnostic tools and antibiotic treatments [5]. Furthermore, the growing emergence of multidrug-resistant (MDR) and extensively drug-resistant (XDR) strains underscores the need for real-time, high-resolution surveillance tools capable of tracking transmission and resistance evolution within healthcare institutions [6]. MDR is frequently associated with isolates belonging to these global clones [7,8]. This need is particularly urgent given that antimicrobial resistance (AMR) remains a global public health threat and is a leading cause of long-term hospitalization, increased morbidity and mortality, and escalating healthcare costs [9]. Hospitals have traditionally been seen as the primary reservoirs of AMR infections, but an increasingly urgent concern is the spread of antimicrobial-resistant bacteria within communities and the environment [10,11]. While often associated with healthcare environments, initial infections can also stem from individuals arriving from external facilities. The ability of *A. baumannii* to develop resistance to multiple antimicrobial agents, along with its persistence on environmental surfaces for extended periods, highlights its role as a leading cause of nosocomial infections [12,13]. The widespread use of antimicrobials is the principal driver of AMR, both within and beyond hospital settings [14]. Studies have demonstrated that communities can act as a key reservoir for antimicrobial-resistant bacteria [15,16]. Infection prevention measures, particularly enhanced surveillance and control protocols, are crucial to combating the spread of AMR [17,18]. Moreover, the emergence of hypervirulent strains complicates treatment and control efforts, necessitating the continuous adaptation of strategies to combat this evolving threat [19]. The rise in MDR strains has underscored the need for a deeper understanding of their genomic characteristics, phylogenetic relationships, and epidemiological distribution in order to improve infection surveillance and control strategies. Genomic and epidemiological approaches, supported by bioinformatics workflows, have emerged as powerful tools to characterize *A. baumannii* strains at a molecular level. Whole-genome sequencing (WGS) enables high-resolution analysis of bacterial genomes, allowing for precise strain identification, AMR profiling, and the detection of virulence factors [20]. Phylogenetic analysis is a valuable tool for tracing evolutionary relationships among organisms through the examination of genetic sequences, typically focusing on core genes or entire genomes [18]. When applied to MDR *A. baumannii*, phylogenetic analysis can elucidate transmission dynamics, identify clonal lineages, and track the spread of resistance genes [18]. Moreover, comparative genomics combined with phylogenetic analysis offers critical insights into the evolutionary relationships and transmission pathways of MDR *A. baumannii* in both clinical and environmental settings.

This study addresses these gaps by performing an integrated genomic and epidemiological investigation of 44 MDR *A. baumannii* isolates collected across different wards within an Italian hospital. Through a comprehensive genomics and bioinformatics workflow, including WGS, MLST, phylogenetic reconstruction, pan-genome analysis, and the detection of AMR and virulence genes, this study aims to

1. Characterize clonal diversity and resistance profiles at the hospital scale;
2. Uncover potential transmission clusters and ward-specific genomic signatures;
3. Inform surveillance strategies by integrating genomic data with clinical and epidemiological metadata.

By bridging molecular data with hospital-level information, this work provides actionable insights to support targeted infection prevention and antimicrobial stewardship efforts within healthcare settings.

## 2. Materials and Methods

### 2.1. Bacterial Isolates

Forty-four *A. baumannii* strains were isolated from clinical infections occurring between 2022 and 2023 in patients admitted to various wards of the Fondazione Policlinico Universitario Campus Bio-Medico in Rome, Italy. The strains were selectively isolated from colonies grown on McConkey agar and identified using MALDI-TOF (Bruker Daltonics GmbH, Bremen, Germany). Antimicrobial susceptibility testing was performed using a semi-automated broth dilution method and a disk diffusion method. Cefiderocol disks (FDC, antibiotic disk 30 µg, Liofilchem, Roseto degli Abruzzi, Italy) were applied to the surface of a culture medium inoculated with a pure colony suspension to the density of a 0.5 McFarland of Acinetobacter baumannii. After incubation, the plates were examined and the inhibition halos around each disk were compared with the standard inhibition haloes, according to the Eucast guidelines using a Sensititre Gram-Negative DKMGN Plate (Thermo Fisher Scientific, Waltham, Massachusetts, Stati Uniti) (broth microdilution according to ISO standard for Colistin [21,22]. For each isolate, information was collected on the infection site, hospital ward, and year of isolation, and antibiotic resistance patterns were recorded, as summarized in Table 1. This study was conducted according to the guidelines of the Declaration of Helsinki and approved by the Ethical Committee of the Fondazione Policlinico Universitario Campus Bio-Medico of Rome (28.16 TS Com Et CBM). Informed consent was not required due to the retrospective design of the study.

### 2.2. DNA Extraction and Whole-Genome Sequencing (WGS)

Genomic DNA was extracted from a single colony grown on Columbia blood agar at 37 °C using PowerBead Tubes, Glass 0.5 mm (Qiagen, Hilden, Germany), and the Illumina DNA Prep workflow (Illumina, San Diego, CA, USA) following the manufacturer’s instructions. Libraries were prepared and sequenced on an Illumina MiSeq platform with a specific read length (Illumina, San Diego, CA, USA).

### 2.3. Bioinformatics Analysis

Bioinformatic analysis of the 44 *A. bumannii* isolates followed the pipeline defined by the rMAP v1.0 tool [23], with modifications to incorporate updated versions of specific toolsRaw FastQ reads trimmed using Trimmomatic v0.39 [24], and quality was assessed with FastQC v0.11.9. The trimmed and filtered reads were then assembled using MEGAHIT v1.2.9 [25] and binned using concoct v1.1.0 [26]. Assembly quality was evaluated with QUAST v5.3.0 [27]. The taxonomic classification of binned genomes was confirmed using GTDB-Tk v2.4.0 [28], and genome quality was assessed via CheckM v1.2.3 [29]. By adopting the criteria established by the Genomics Standard Consortium [30], samples with completeness <50% and contamination ≤10% were excluded from further analysis. Average nucleotide identity (ANI) was calculated using a custom script [31,32], and multilocus sequence typing (MLST) was assigned to each sample using mlst tool v2.19.0 [33]. Phylogenetic trees were generated from the binned genomes using PHAME [34] with *A. baumannii* as the reference genome. RAxML was employed to construct trees with 100 bootstrap replicates. This tool utilizes only the alignment positions that are 100% conserved, yet only single-nucleotide polymorphisms (SNPs) that can be identified in the dataset genomes are present in the reference genome. To complement this, kSNP4.1 [35] was used for reference-free SNP detection across the pan-genome. The analysis was configured with a maximum likelihood model, and Kchooser4 determined the appropriate k-mer size (17 or 21). In order to ascertain the presence of clusters within the strains under analysis, the ClusterPicker1.2 tool [36] was utilized. This tool requires an aligned FASTA file and a phylogenetic tree with bootstrap values. The tree generated by PHAME was used, with parameters set to a genetic distance of 0.15% and 90% bootstrap support. This low genetic distance cut-off is substantiated by the elevated pairwise average nucleotide identity (ANI) value obtained from the analyzed strains. Cluster visualization was performed using FigTree v1.4.4 [37]. Roary v.3.13.0 [38], via the Galaxy Server [39], was used to perform pangenome analysis. Graphical outputs were obtained using additional scripts such as roary_plots.py, roary2svg.pl [40], and Fripan [41]. Antibiotic resistance gene detection was carried out using AMRfinder v4.0.3 [42], combining the (.gff) and (.faa) files generated by prokka v1.14.6 [43]. Annotation files were added to produce the most informative output. To avoid reporting common point mutation for the species, *A. Baumannii* was specified as an organism option. Virulence factor and toxin predictions were performed using PathoFact v1.0 [44], which combines HMM domain databases with a random forest classifier. It also integrates the SignalP tool to predict secreted proteins. However, this tool offered only a quantitative prediction of virulence factors; consequently, to further refine the characterization of virulence factors, MetaVF [45] was used, as it is based on an expanded version of the Virulence Factor Database (VFDB). A heatmap was created using ComplexHeatmap v2.20.0 [46] in R 4.4.1 [47] to visualize resistance genes and secreted/non-secreted toxins. The ggstatsplot v0.12.3 [48] library was used to test the differential distribution of antibiotic resistance genes between the hospital wards. Phylogenetic tree charts were generated via the ggtreeExtra v1.10.0 [49] and ggtree v3.8.2 [50] libraries.

## 3. Results

### 3.1. Isolates

The strains were isolated from various clinical samples collected from patients admitted to different wards of the hospital between 2022 and 2023, as reported in Table 1.

Of the 44 patients, *A. baumannii* was isolated from blood cultures in nine cases (20%), indicating bloodstream infection infections. In three cases each (7%), the pathogen was isolated from broncho-alveolar lavage (BAL) samples and in four cases (9%) was isolated from wound swabs. Regarding drainage fluid, peritoneal fluid, ulcers, and pleural fluid, one case was identified for each infection site (2%). Additionally, in five cases (11%), the organism was recovered from both rectal swabs and urine cultures. *A. baumannii* was also identified in sputum samples in eight cases (18%) and in tracheal aspirates in seven cases (16%) (Table 2).

Forty-seven strains of multi-drug resistant (MDR) and carbapenem-resistant Acinetobacter baumannii (CRAB) were selected; 86% of the strains were resistant to all antibiotics tested with the automatic Phoenix instrument, and the remaining 14% presented the same resistances except for sensitivity to amikacin. Colistin was tested for all strains using a broth microdilution method; only 2/47 (4%) strains showed resistance to the antibiotic. Cefiderocol was tested using an agar diffusion method (the EUCAST standard disk diffusion method); 8 strains out of 47 show an inhibition zone <17 mm. For this antibiotic there is insufficient clinical data to determine a clinical breakpoint. Isolates with MIC values ≤0.5 mg/L (zone diameter ≥21 mm) are mostly devoid of resistance mechanisms. Isolates with MICs of 1–2 mg/L have acquired resistance mechanisms which may result in impaired clinical response. Isolates with MIC values >2 mg/L (zone diameter <17 mm) will likely be resistant (EUCAST “The European Committee on Antimicrobial Susceptibility Testing. Breakpoint tables for interpretation of MICs and zone diameters Version 15.0, 2025. https://www.eucast.org”) (Table 3).

### 3.2. Quality Control and Assembly Process

The quality control process revealed that all 44 samples exhibited high sequencing quality, with a mean quality value greater than 30 across the entire read length and an average of 1.3 million reads per sample. The average number of contigs detected in the samples is 396 (range: 185–615), with an average coverage of 77X (range: 27X to 97X) and an average N50 of 116,477 (range: 72,961 to 165,154). The number of genome bins per sample varied from a minimum of 17 to a maximum of 82, with an average of 59 bins (Supplementary Table S1—Assembly Report). Genomes identified as *Acinetobacter* but not meeting the minimum quality requirements (completeness <50% and contamination ≤10%) were discarded, reducing the dataset to 41 samples (Supplementary Table S1—CheckM). Subsequently, a taxonomic assignment process was conducted on each binned metagenome, thereby confirming their classification as *A. baumannii* (Supplementary Table S1—GTdbTK). Once reliable sequencing data were obtained, it was possible to arrive at the overall pairwise similarities of the isolated *A. baumannii* using the average nucleotide identity (ANI) value. The ANI ranged between 98% (comparing SAMPLE-34 with both SAMPLE-43 and SAMPLE-38) to 99%, which indicates a close genomic relationship.

### 3.3. MLST and Phylogenetic Trees

**Figure 1:**
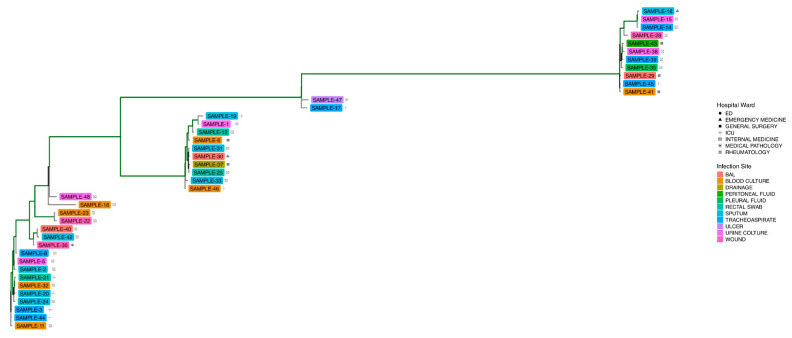
Phylogenetic representation of *Acinetobacter baumannii* strains, excluding SAMPLE-34, that visually supports the presence of clusters. The tree branches highlighted in green have a support ≥ 0.9.

**Figure 2:**
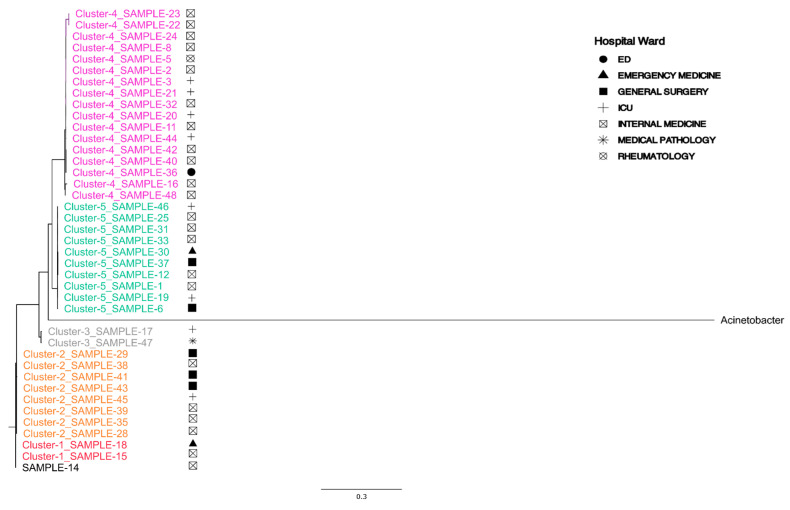
Phylogenetic reconstruction of *A. baumannii* strains with highlighted clusters.

MLST classification of the assembled genomes revealed that all samples belonged to ST2, with the exception of only SAMPLE-34, which was classified as ST1 (Supplementary Table S1—MLST). Phylogenetic analysis conducted using the PhaME tool demonstrated that SAMPLE-34 exhibited a greater phylogenetic distance from the other isolates (Figure S1). This distance was further supported by the kSNP4 analysis (Figure S2). Excluding SAMPLE-34 from the PhaME and kSNP4 analysis enables the identification of additional variations within the strains under investigation. The analysis of both phylogenetic trees revealed the presence of four distinct clusters (Figure S3). These clusters were particularly evident in the KSNP4 product tree, which can be attributed to the absence of the reference genome of *A. baumannii* (Figure 1). The analysis shows that no clusters are predominantly associated with hospital ward or site of infection. The only identifiable clusters are those based on phylogenetic distance so, to ensure accuracy, it was first necessary to verify the clusters identified visually in Figure 1 through the ClusterPicker computational tool. Application of ClusterPicker tool on the RAxML tree produced by PhaME confirmed the presence of these clusters, with a few minor exceptions. Specifically, SAMPLE-14 and SAMPLE-34 did not fall into any identified cluster (assigned as Cluster “−1”), while SAMPLE-15 and SAMPLE-18 formed a separate cluster of their own (Figure 2). 

It is important to note that Cluster 4 was predominantly composed of samples from internal medicine and intensive care units (ICUs), with the exception of SAMPLE-36, which originated from the Emergency Department (ED). Concurrently, SAMPLE-47 and SAMPLE-17 exhibited a distinct grouping, clustering separately between Cluster 2 and Cluster 5 (Figure 1 and Figure 2). 

### 3.4. Pan-Genome Analysis

**Figure 3:**
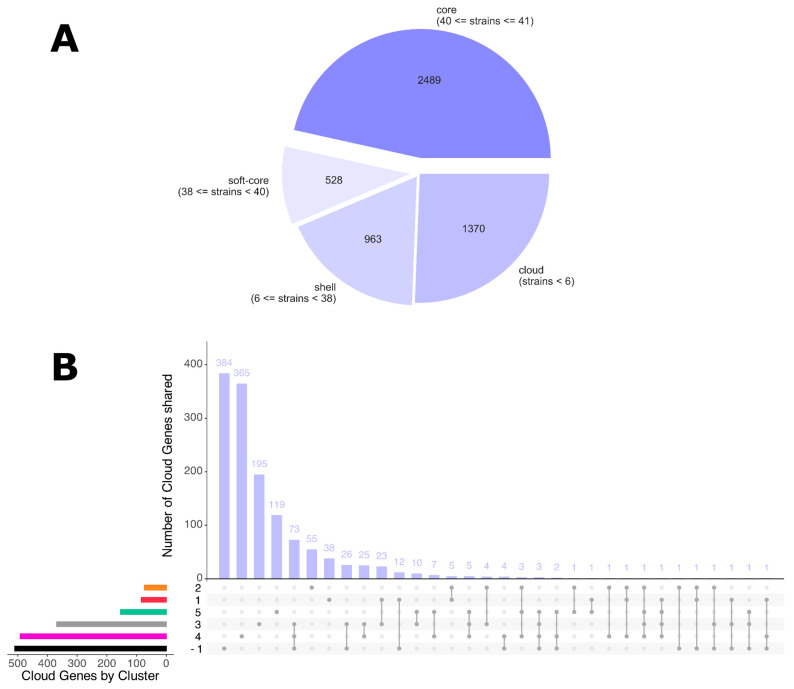
(**A**) The figure illustrates the proportion of core genes shared by the majority of the cases analyzed (40 ≤ strains ≤ 41), soft-core genes shared by a significant portion of the cases (38 ≤ strains < 40), shell genes (6 ≤ strains < 38), and cloud genes present in fewer than six strains among the cases analyzed. (**B**) Cloud genes are analyzed in detail with respect to the clusters identified at the phylogenetic level; the bar chart shows, in descending order, the number of cloud genes specific to individual clusters and shared between clusters. The horizontal bar plot at the bottom left corresponds to the number of genes per cluster: black bar cluster −1, fuchsia bar cluster 4, grey bar cluster 3, green bar cluster 5, red bar cluster 1 and orange bar cluster 2.

The pan-genome composition revealed a total of 5050 genes, being categorized as follows: 2489 genes are designated as ‘core’ (present in a minimum of 40 strains), 528 are classified as ‘soft-core’ (present in a minimum of 38 and a maximum of 39 strains), 963 are categorized as ‘shell’ (present in less than 38 strains but at least 6 strains), and 1370 are termed ‘cloud’ (present in less than 6 strains) (Figure 3A). Detailed analysis of cloud genes shows more gene sharing between Cluster −1, Cluster 4, and Cluster 3 than between the others (n shared genes = 73) (Figure 3B). Excluding all “hypothetical proteins,” it is noteworthy that these clusters share proteins involved in the formation of Type IV Secretion Systems (T4SS), virulence factors, and resistance to tetracycline (gene tetA) (Supplementary Table S1—Genes Common to Cluster −1, Cluster 3, and Cluster 4). The tetA gene, which confers resistance to tetracycline, is present in SAMPLE-17, SAMPLE-34, SAMPLE-47, and SAMPLE-48. In addition, the traC and traD genes that are associated with T4SS are present in SAMPLE-17, SAMPLE-34, SAMPLE-36, SAMPLE-40, SAMPLE-42, and SAMPLE-47. These findings suggest the potential for horizontal exchange of virulence factors and antibiotic resistance among these strains. Figure S4 illustrates the distribution of gene presence across the various samples, showing that while Cluster 4 is subdivided into two sub-clusters, the remaining clusters are distinctly distinguishable. The marginal gene composition variation that led to the differentiation of the cluster was already revealed by the phylogenetic reconstruction (Figure 1). In the current reconstruction, samples SAMPLE-48, SAMPLE-16, SAMPLE-23, SAMPLE-22, SAMPLE-40, SAMPLE-42, and SAMPLE-36 revealed slightly higher phylogenetic distances from the remaining Cluster 4. The SAMPLE-34 reflects a cluster of highly specific genes (Supplementary Table S1—SAMPLE-34 Specific genes), supporting its distinct phylogenetic placement. Out of the 383 strain-specific genes within SAMPLE-34, 206 have a biological annotation and 80 correspond to virulence factors, horizontal gene transfer, antibiotic resistance genes and potential antimicrobial targets. This renders SAMPLE-34 highly specific, both phylogenetically, and with regard to virulence factors and antibiotic resistance profiles.

Multidimensional Scaling (MDS) analysis based on gene presence/absence further confirmed the clustering patterns (Figure S5). The most clearly distinguishable clusters are Cluster 2 (including Cluster 1) and Cluster 5, while Cluster 4 displays greater heterogeneity. SAMPLE-47 and SAMPLE-17, part of Cluster 3, are positioned further away from the main clusters, demonstrating a greater degree of divergence. 

### 3.5. Gene Resistance Analysis

**Figure 4:**
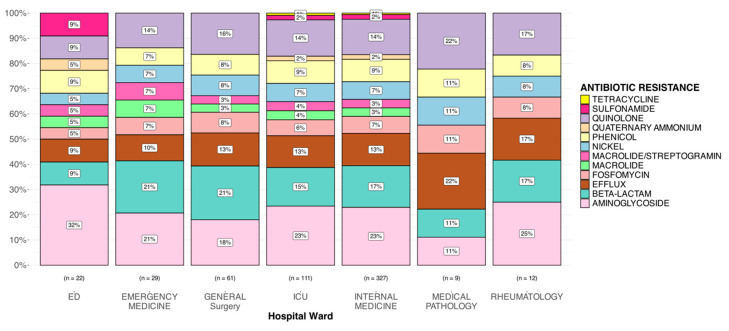
Gene resistance analysis.

The analysis then focused on identifying the genes conferring the antibiotic resistance mutations in the different samples (Figure 4). Pearson’s χ^2^ test was used to evaluate whether the distribution of resistance genes differed significantly between hospital wards. Although the overall test did not yield statistically significant results, indicating that the distribution of antibiotic resistance is not ward-specific. However, within the ICU (*p* = 1.42 × 10^−10^), General Surgery (*p* = 5.61 × 10^−6^), and Internal Medicine (*p* = 1.26 × 10^−38^), there was a notable imbalance in the distribution of genes associated with antibiotic resistance. Particularly, there was a higher proportion of genes conferring antibiotic resistance in Aminoglycoside, Beta-lactam, Efflux, and Quinolone. The lack of overall statistical significance may be due to the low number of genes conferring antibiotic resistance in some wards, such as the ED (*n* = 22), Emergency Medicine (*n* = 29), Medical Pathology (*n* = 9), and Rheumatology (*n* = 12) wards. Additionally, Quaternary Ammonium and Sulfonamide resistance was observed only in the ED, ICU, and Internal Medicine. Tetracycline resistance was exclusive to isolates from Internal Medicine and the ICU. The analysis was also conducted in quantitative terms, with the number of antibiotic resistance genes, virulence factors and toxins being examined in relation to hospital ward, infection site, and genetic clusters. The results of these comparisons indicated that there was no significant difference in the number of antimicrobial resistance (AMR) genes, virulence factors, or toxins among the studied groups (Supplementary Table S2). Genetic clusters remain the most effective method of strain separation in the study, and as such, they will be the primary focus of this discussion.

**Figure 5:**
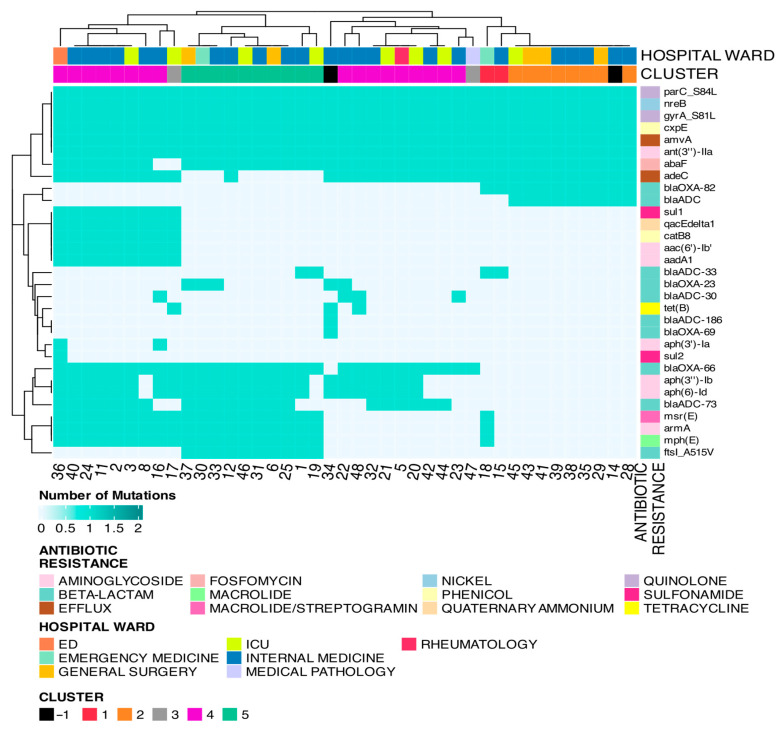
Heatmap of presence of AMR-conferring mutations for each patient.

The heatmap (Figure 5) illustrates the presence of antibiotic-resistance-associated mutations in each patient. Based on these profiles, four groups can be distinguished. The first group includes nine samples, mostly from Cluster 4, sharing multiple resistance genes. The second group contains 10 samples from Cluster 5 with a distinct yet consistent resistance pattern. The third group, also from Cluster 4, includes 10 samples with a differing resistance signature. The fourth group consists of nine samples belonging to Cluster 2. The most evident distinction that results in the separation of strains classified in Cluster 4 with respect to antibiotic resistance genes is the occurrence of mutations that determine resistance to quaternary ammonium (*qacEdelta1* gene), phenol (*catB8* gene), and aminoglycosides (*aac(6′)-lb′* and *aadA1* genes). SAMPLE-15 and SAMPLE-18 display resistance profiles similar to Cluster 2 but also exhibit notable differences. SAMPLE-18, in particular, shares AMR genes such as *msr(E)*, *armA,* and *mph(E)* with samples from Cluster 4 and Cluster 5. Both samples also possess mutations in the *BlaADC-33* gene, conferring beta-lactam resistance, while lacking resistance in the *BlaADC* gene. These differences justify the assignment of SAMPLE-15 and SAMPLE-18 to a distinct group, Cluster 1, despite their genetic proximity to Cluster 2. On the other hand, with regard to SAMPLE-14, which was not assigned to any cluster, it is not possible to distinguish it from Cluster 2 in terms of antibiotic resistance. Finally, SAMPLE-34 stands out due to the presence of three specific genes: *tet(B)*, *blaOXA-66*, and *blaADC-186*. The latter two genes contain mutations conferring beta-lactam resistance, while *tet(B)* confers resistance to tetracycline. It is noteworthy that the resistance to tetracycline exhibited by SAMPLE-34 had previously been examined in the section on pangenome analysis concerning the tetA gene. The protein detected in the pangenome analysis is “Tetracycline resistance protein, class B.” At the time of publication, only “tetracycline efflux ABC transporter TetAB subunit A” was present in the database of the AMRFinderPlus software (https://ftp.ncbi.nlm.nih.gov/hmm/NCBIfam-AMRFinder/latest/, 29 September 2025). Therefore, the use of this software could not validate the hypothesis for this antibiotic resistance acquisition.

### 3.6. Virulence Factors and Toxin Analysis

**Figure 6:**
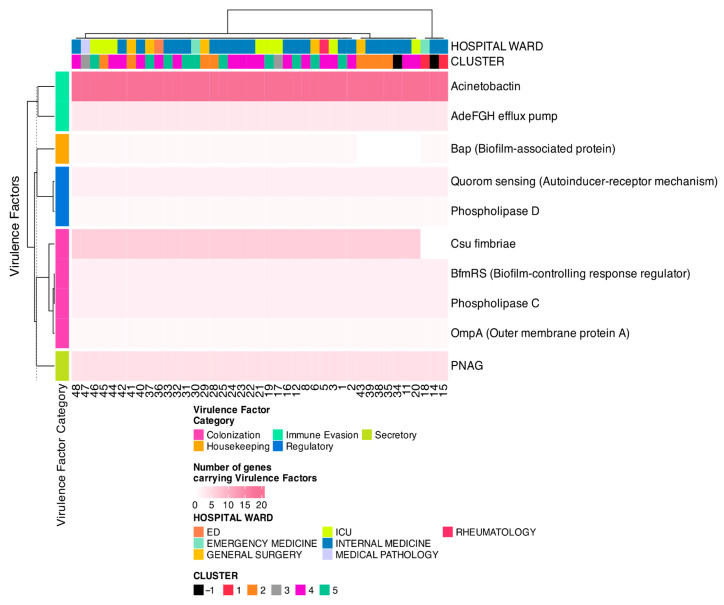
Heatmap of presence of virulence factors each patient.

Finally, the prediction of virulence factors and toxins by PathoFact showed that the number of virulence factors was comparable between the different samples (Figure S6). On average, each sample contained 514 non-secreted virulence factors (range: 484–549), and 255 secreted virulence factors (ranging from 240 in SAMPLE-18 to 278 in SAMPLE-42) (Figure 6). MetaVF analysis confirmed that all identified virulence genes corresponded to *A. Baumannii*, with 381 out of 400 (i.e., 95.25%) being 100% complete. Of the 41 strains, 31 carried all 40 virulence factors, 7 carried 39, and 3 (SAMPLE-14, SAMPLE-15, and SAMPLE-18) carried 34, corresponding to a completeness of 90–100%, except for SAMPLE-34, which showed only 53.84%. The tool indicates that the genes “Csu fimbriae” and “BfmRS” (Biofilm-Controlling Response Regulator) are present in plasmids. The majority of genes are characterized by the presence of virulence factors that are associated with the ‘Immune Evasion’ category and “Acenovactin” in particular is the main virulence factor (Figure 6). While virulence profiles did not allow for clear discrimination between clusters, SAMPLE-14, SAMPLE-15, and SAMPLE-18 were distinguishable due their lack of ‘Csu fimbriae’, a key biofilm-associated gene.

**Figure 7:**
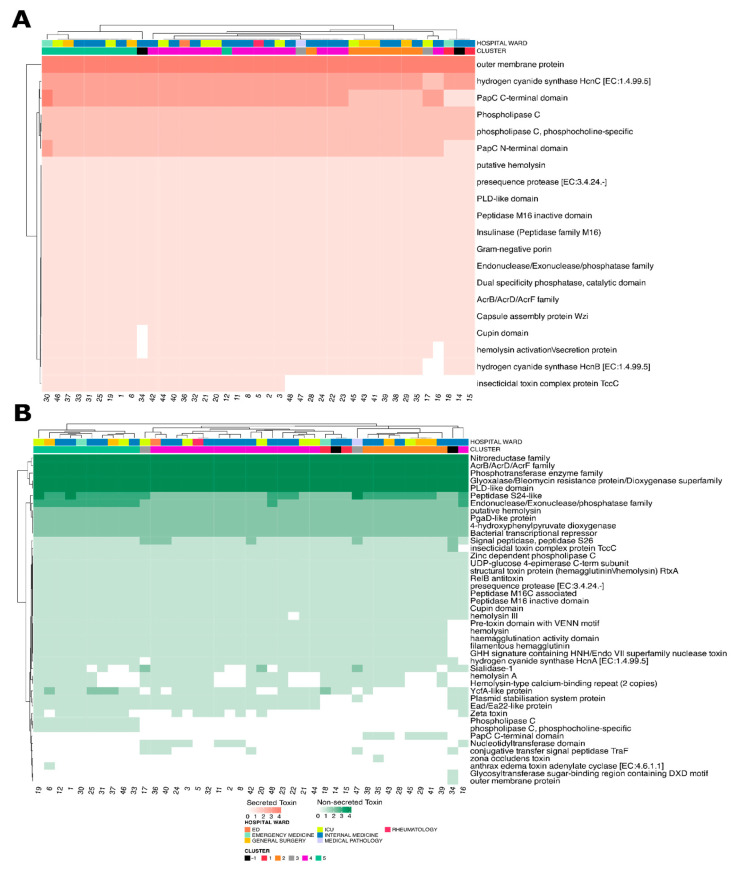
(**A**) The figure illustrates the distribution of toxins secreted in the strains analyzed. For each strain, the hospital department of origin and the phylogenetic cluster are reported. (**B**) The figure illustrates the distribution of toxins that were not secreted in the analyzed strains. For each strain, the hospital department of origin and the phylogenetic cluster are reported.

Analysis of secreted toxins showed a generally uniform distribution across samples (Figure 7A), although the ‘Insecticidal Toxin Complex Protein TccC’ was absent in strains from Cluster 1, Cluster 2, and several from Cluster 4. Interestingly, SAMPLE-12 and SAMPLE-28 (Cluster 5) displayed high toxin profile similarity with strains from Cluster 4, while SAMPLE-16 (Cluster 4) showed closer resemblance to Cluster 1 strains. This absence appeared to be nearly complete in Clusters 1 and 2, while in Cluster 4 a few isolates retained partial representation, suggesting non-uniform loss. Such a pattern may indicate either evolutionary divergence in toxin repertoires or selective adaptation mechanisms. Interestingly, SAMPLE-12 and SAMPLE-28, which are formally assigned to Cluster 5, displayed a toxin domain profile closely resembling that of Cluster 4 strains (Figure 7A,B). This similarity was not limited to overall clustering but appeared to be driven by shared absence/presence patterns across specific toxin domains, including the reduced representation of TccC. This suggests that despite their cluster designation, these isolates may functionally align more closely with Cluster 4. Another remarkable finding was the deviation of SAMPLE-16 from the characteristic profile of its assigned cluster. While grouped within Cluster 3, this isolate displayed a marked reduction in toxin domain representation (Figure 7B). Quantitatively, SAMPLE-16 lacked at least two domains that were otherwise consistently present in Cluster 3 strains, resulting in a decreased predicted repertoire of secreted toxins. This atypical profile may reflect functional attenuation.

For non-secreted toxins (Figure 7B), fewer distinct patterns were observed. However, the Nucleotidyltransferase domain, which had been exclusively present in the Cluster 4 samples and phospholipase C (including the phosphocholine-specific form), was found only in Cluster 5. It is evident that clusters are predominantly maintained, with the exception of SAMPLE-16, which deviated from its designated cluster by having a significantly lower number of secreted toxins in comparison to the other strains.

## 4. Discussion

*Acinetobacter baumannii* is an emerging and serious pathogen responsible for nosocomial infections in humans [51,52,53,54]. Precise identification and species-level differentiation of *A. baumannii* remains challenging. While MALDI-TOF is a standard identification method with scores ≥ 2.3 indicating “highly probable species identification” scores ranging from 2.0 to 2.29 suggest a “secure genus identification and probable species identification” [55]. Currently available phenotypic techniques, including standard biochemical methods and automated systems, may not be always sufficient [56], and require technologies that can provide deeper insights. Thus, tools and high-resolution typing methods are essential for reliable identification and epidemiological analysis [57,58]. In this study, we characterized *A. baumannii* strains isolated from various infection sites and hospital wards between 2022 and 2023. Our goals were to explore their genomic diversity, phylogenetic relationships, antibiotic resistance profiles, and virulence factors. Bloodstream infections were the most common (20%), followed by sputum (18%) and tracheal aspirate samples (16%). The pathogen was also isolated from BAL, urine cultures, wounds, and rectal swabs, indicating its capacity to colonize diverse anatomical sites. Its presence in rectal swabs supports its potential role as a colonizer and reservoir in hospital settings [59,60,61]. The sequencing and assembly process confirmed high-quality genomic data and, after filtering out low-quality assemblies, 41 samples were retained for further analysis. The high degree of genomic similarity suggests a close evolutionary relationship among the strains, possibly indicating nosocomial transmission or a common source of infection. MLST is a method used to describe bacterial populations and is considered the gold standard of typing, as it analyzes specific housekeeping genes to determine the phylogenetic relationship between bacterial strains [62]. Our MLST analysis classified all the samples as ST2, but only SAMPLE-34 was classified as ST1, suggesting genetic divergence likely due to recombination, mutation, or the acquisition of exogenous elements [63]. The unique phylogenetic position of SAMPLE-34 suggests a divergent evolutionary lineage was further supported by both PhaME and kSNP4 analyses. ST2 belongs to international clone II and is the most dominant type globally [64]. When we looked at the phylogenetic analysis from a departmental perspective, four distinct clusters emerged, composed mainly of strains from internal medicine and intensive care units. SAMPLE-34 was isolated from a patient admitted to the internal medicine ward after presenting to the emergency department in April 2024 with symptoms including a fever of 38 °C and lower limb edema with diabetic ulcers. The patient’s medical history shows a previous hospitalization in the same hospital and ward within the preceding two months for mild exertional dyspnea, dependent edema, and abdominal pain associated with generalized pruritus, from which the patient was discharged in February 2024. These distinct clusters may suggest that multiple strains of *A. baumannii* are circulating within the hospital, increasing and contributing to infections that become persistent and pose new therapeutic challenges. We further investigated this by performing a pan-genomic analysis, which allowed us to analyze the genetic set of an entire species, including all the genes present in its strains. This analysis identified a total of 5050 genes, including 2489 core genes, 528 soft-core genes, 963 shell genes, and 1370 cloud genes, suggesting that the pan-genome of these bacterial isolates is quite large. In particular, the presence of a significant number of shell genes (i.e., the remaining genes present in several genomes) and cloud genes (i.e., rare genes present only in one/two genomes) reflects a considerable genetic variability among strains, potentially due to gene acquisition mechanisms such as horizontal gene transfer (HGT) or adaptive mutations. In fact, shell and cloud form the accessory component of the genome that contributes to species diversity and can confer selective advantages, such as niche adaptation, antibiotic resistance or colonization of a new host [65,66]. Instead, core genes represent the backbone of all strains of a given species and the set of genes essential for the survival of the bacterium and probably encode for fundamental functions such as central metabolism, DNA replication, and resistance to environmental stress, and are responsible for the main phenotype [67]. Soft-core genes, although present in most isolates, may provide specific selective advantages to certain strains, influencing virulence or antibiotic resistance [65]. The distribution of genes in the clusters suggested considerable genetic variability, with SAMPLE-34 showing a unique gene profile, confirming the previous finding. Its distinctive gene profile suggests that this isolate may have acquired genetic characteristics from other bacteria present in the hospital environment or from mutations that confer specific adaptive capabilities, or it may have lost genes compared to the other strains analyzed. The presence of specific genes in distinct clusters may indicate adaptive strategies that allow for survival in different hospital environments, such as the ability to resist disinfectants and antibiotics or to persist on inanimate surfaces [68,69]. Furthermore, specific genes in the shell and cloud clusters could be related to mechanisms of antibiotic resistance, biofilm formation, or the evasion of the host immune system, conferring a selective advantage in high selection pressure environments such as hospitals [70]. When assessing antibiotic resistance genes, their presence was widespread in the samples, with significant variations between different hospital departments. We highlighted that among the most widespread resistance genes were those associated with resistance to aminoglycosides, beta-lactams, efflux pumps, and quinolones [71,72]. We also evaluated the trend of resistance genes appearing in certain hospital wards, and it emerged that the Intensive Care, General Surgery, And Internal Medicine wards showed a higher frequency of antibiotic resistance genes, probably due to the selective pressure resulting from the prolonged use of antibiotics. In particular, SAMPLE-34 harbored three unique resistance genes (tet(B), blaOXA-66, and blaADC-186), reinforcing its distinct genetic background. To gain a deeper insight into the bacterium, we analyzed virulence factors, which revealed the overall conservation of immune evasion mechanisms. However, SAMPLE-14, SAMPLE-15, and SAMPLE-18 lacked Csu fimbriae, a key biofilm-related gene. This gene, along with BfmRS, was detected on plasmids, underscoring its potential role in persistent infections [73,74]. In particular, SAMPLE-14, SAMPLE-15, and SAMPLE-18 showed a distinct virulence factor profile, lacking the Csu fimbriae gene. Toxin analysis also demonstrated a largely conserved distribution, although some non-secreted toxins, such as phospholipase C, were specific to Cluster 5, while PapC domain proteins were exclusive to Cluster 4. Phospholipase C contributes to the virulence and haemolytic activity of bacteria; in fact it is involved in the metabolism of phospholipids and can influence the stability of epithelial cell membranes [75,76].

These findings have potential implications for hospital infection control and decision-making. The identification of specific clonal clusters with high resistance and virulence burdens, particularly in the ICU and Internal Medicine wards, may support the implementation of targeted screening policies upon patient admission in high-risk units. Moreover, genomic surveillance could inform alert systems in the microbiology laboratory, flagging the re-emergence of high-risk clones (e.g., those harboring multiple AMR genes or lacking key virulence regulators such as Csu fimbriae). Genomic data could also guide empirical therapy in wards where certain resistance profiles are prevalent and support preemptive isolation of colonized patients to prevent transmission.

## 5. Conclusions

Thus, our study highlighted the genomic diversity and antibiotic resistance patterns of *A. baumannii* isolates collected from different hospital settings. Our results provide a framework for genotype- and phenotype-guided therapeutic strategies, enabling more personalized and effective treatment approaches. Furthermore, phylogenetic analysis identified distinct clonal clusters, suggesting the presence of multiple circulating lineages, which may contribute to persistent nosocomial infections. The substantial presence of antibiotic resistance genes demonstrates the need for stringent infection control measures and targeted antibiotic stewardship programs. Overall, our results suggest that the marked genetic variability of *A. baumannii* is a key factor in its ability to adapt and persist in hospital environments, and in the increasing risk of the dissemination of highly resistant and virulent strains.

## 6. Perspectives for Future Studies

Further research should aim to identify specific genetic determinants associated with hospital survival, and assess their potential correlation with nosocomial outbreaks. Transcriptomic analyses, particularly on outlier strains such as SAMPLE-34, could reveal the functional role of unique genes in relation to pathogenicity and antimicrobial resistance. Additionally, comparative analyses with curated resistance and virulence gene databases may help identify novel therapeutic targets, potentially contributing to the development of new therapeutic strategies to counter infections caused by emerging strains of *A. baumannii*.

Future studies will take into consideration these aspects, which have not been explored in this work:

1. Expanding the number of isolates and including samples from multiple institutions to improve the generalizability of findings.
2. Improving detailed clinical metadata such as patient outcomes, antibiotic exposure, or length of hospital stay, which could have enhanced interpretation of resistance and virulence findings.
3. Refining clonal classification methods, as the current use of fixed genetic distance thresholds may not fully align with epidemiologically meaningful transmission units.

By addressing these aspects, future studies can provide a more comprehensive understanding of the evolution, transmission, and clinical relevance of *A. baumannii* in healthcare environments.

## Figures and Tables

**Table 1:** Isolate descriptions: ID samples, hospital ward, site of infection, and date of isolation.

ID Samples	Hospital Ward	Site of Infection	Date of Isolation
SAMPLE-1	Internal Medicine	Urine culture	27 December 2022
SAMPLE-2	Internal Medicine	Sputum	8 January 2023
SAMPLE-3	ICU	Tracheoaspirate	11 January 2023
SAMPLE-5	Rheumatology	Urine culture	15 January 2023
SAMPLE-6	General Surgery	Blood culture	17 January 2023
SAMPLE-7	ICU	Broncho-alveolar lavage (BAL)	26 January 2023
SAMPLE-8	Internal Medicine	Tracheoaspirate	1 February 2023
SAMPLE-11	Internal Medicine	Blood culture	16 February 2023
SAMPLE-12	Internal Medicine	Rectal swab	22 February 2023
SAMPLE-14	Internal Medicine	Tracheoaspirate	24 February 2023
SAMPLE-15	Internal Medicine	Urine culture	27 February 2023
SAMPLE-16	Internal Medicine	Blood culture	1 March 2023
SAMPLE-17	ICU	Tracheoaspirate	5 March 2023
SAMPLE-18	Emergency Medicine	Sputum	6 March 2023
SAMPLE-19	ICU	Sputum	6 March 2023
SAMPLE-20	ICU	Sputum	6 March 2023
SAMPLE-21	ICU	Rectal swab	6 March 2023
SAMPLE-22	Internal Medicine	Wound	11 March 2023
SAMPLE-23	Internal Medicine	Blood culture	13 March 2023
SAMPLE-24	Internal Medicine	Sputum	14 March 2023
SAMPLE-25	Internal Medicine	Rectal swab	19 March 2023
SAMPLE-26	Internal Medicine	Rectal swab	19 March 2023
SAMPLE-27	Internal Medicine	Blood culture	21 March 2023
SAMPLE-28	Internal Medicine	Wound	22 March 2023
SAMPLE-29	General Surgery	Bal	23 March 2023
SAMPLE-31	Internal Medicine	Sputum	27 March 2023
SAMPLE-32	Internal Medicine	Blood culture	29 March 2023
SAMPLE-33	Internal Medicine	Sputum	30 March 2023
SAMPLE-34	Internal Medicine	Wound	4 April 2023
SAMPLE-35	Internal Medicine	Pleural fluid	5 April 2023
SAMPLE-36	ED	Wound	5 April 2023
SAMPLE-37	General Surgery	Drainage	7 April 2023
SAMPLE-38	Internal Medicine	Urine culture	11 April 2023
SAMPLE-39	Internal Medicine	Tracheoaspirate	11 April 2023
SAMPLE-40	Internal Medicine	Bal	13 April 2023
SAMPLE-41	General Surgery	Blood culture	14 April 2023
SAMPLE-42	Internal Medicine	Sputum	22 April 2023
SAMPLE-43	General Surgery	Peritoneal fluid	22 April 2023
SAMPLE-44	ICU	Tracheoaspirate	23 April 2023
SAMPLE-45	ICU	Tracheoaspirate	23 April 2023
SAMPLE-46	ICU	Blood culture	28 April 2023
SAMPLE-47	Medical Pathology	Ulcer	3 May 2023
SAMPLE-48	Internal Medicine	Urine culture	4 May 2023

**Table 2:** Percentage of isolates found by type of infection site.

Site of Infection	Number of Patients	Percentage (%)
BAL	3	7
Blood culture	9	20
Drainage	1	2
Peritoneal fluid	1	2
Pleural fluid	1	2
Rectal swab	5	11
Sputum	8	18
Tracheoaspirate	7	16
Ulcer	1	2
Urine culture	5	11
Wound	4	9

**Table 3:** Antimicrobial susceptibility test profiles.

	PHOENIX								SENSITITRE	DISK DIFFUSION
	MIC (Interpretation)	MIC (Int.)	MIC (Int.)	MIC (Int.)	MIC (Int.)	MIC (Int.)	MIC (Int.)	MIC (Int.)	MIC (Int.)	mm (Int.)
	Amikacin	Ciprofloxacin	Gentamicin	Imipenem	Levofloxacin	Meropenem	Tobramycin	Co-trimoxazole	Colistin	Cefiderocol
SAMPLE	AN	CIP	GM	IPM	LVX	MER	NN	SXT	CL	FDC
SAMPLE 1	>16 (R)	>1 (R)	>4 (R)	>8 (R)	>1 (R)	>16 (R)	>4 (R)	>4/76 (R)	0.5 (S)	23 (S)
SAMPLE 2	>16 (R)	>1 (R)	>4 (R)	>8 (R)	>1 (R)	>16 (R)	>4 (R)	>4/76 (R)	1 (S)	22 (S)
SAMPLE 3	>16 (R)	>1 (R)	>4 (R)	>8 (R)	>1 (R)	>16 (R)	>4 (R)	>4/76 (R)	1 (S)	22 (S)
SAMPLE 5	>16 (R)	>1 (R)	>4 (R)	>8 (R)	>1 (R)	>16 (R)	>4 (R)	>4/76 (R)	1 (S)	23 (S)
SAMPLE 6	>16 (R)	>1 (R)	>4 (R)	>8 (R)	>1 (R)	>16 (R)	>4 (R)	>4/76 (R)	1 (S)	20 (S)
SAMPLE 7	>16 (R)	>1 (R)	>4 (R)	>8 (R)	>1 (R)	>16 (R)	>4 (R)	>4/76 (R)	1 (S)	22 (S)
SAMPLE 8	>16 (R)	>1 (R)	>4 (R)	>8 (R)	>1 (R)	>16 (R)	>4 (R)	>4/76 (R)	1 (S)	23 (S)
SAMPLE 11	>16 (R)	>1 (R)	>4 (R)	>8 (R)	>1 (R)	>16 (R)	>4 (R)	>4/76 (R)	1 (S)	22 (S)
SAMPLE 12	>16 (R)	>1 (R)	>4 (R)	>8 (R)	>1 (R)	>16 (R)	>4 (R)	>4/76 (R)	1 (S)	20 (S)
SAMPLE 14	≤4 (S)	>1 (R)	>4 (R)	>8 (R)	>1 (R)	>16 (R)	>4 (R)	>4/76 (R)	>8 (R)	14 (R)
SAMPLE 15	>16 (R)	>1 (R)	>4 (R)	>8 (R)	>1 (R)	>16 (R)	>4 (R)	>4/76 (R)	8 (R)	13 (R)
SAMPLE 16	>16 (R)	>1 (R)	>4 (R)	>8 (R)	>1 (R)	>16 (R)	>4 (R)	>4/76 (R)	1 (S)	22 (S)
SAMPLE 17	>16 (R)	>1 (R)	>4 (R)	>8 (R)	>1 (R)	>16 (R)	>4 (R)	>4/76 (R)	1 (S)	22 (S)
SAMPLE 18	>16 (R)	>1 (R)	>4 (R)	>8 (R)	>1 (R)	>16 (R)	>4 (R)	>4/76 (R)	0.5 (S)	21 (S)
SAMPLE 19	>16 (R)	>1 (R)	>4 (R)	>8 (R)	>1 (R)	>16 (R)	>4 (R)	>4/76 (R)	1 (S)	15 (R)
SAMPLE 20	>16 (R)	>1 (R)	>4 (R)	>8 (R)	>1 (R)	>16 (R)	>4 (R)	>4/76 (R)	1 (S)	20 (S)
SAMPLE 21	>16 (R)	>1 (R)	>4 (R)	>8 (R)	>1 (R)	>16 (R)	>4 (R)	>4/76 (R)	1 (S)	20 (S)
SAMPLE 22	>16 (R)	>1 (R)	>4 (R)	>8 (R)	>1 (R)	>16 (R)	>4 (R)	>4/76 (R)	2 (S)	22 (S)
SAMPLE 23	>16 (R)	>1 (R)	>4 (R)	>8 (R)	>1 (R)	>16 (R)	>4 (R)	>4/76 (R)	1 (S)	19 (S)
SAMPLE 24	>16 (R)	>1 (R)	>4 (R)	>8 (R)	>1 (R)	>16 (R)	>4 (R)	>4/76 (R)	1 (S)	19 (S)
SAMPLE 25	>16 (R)	>1 (R)	>4 (R)	>8 (R)	>1 (R)	>16 (R)	>4 (R)	>4/76 (R)	1 (S)	22 (S)
SAMPLE 26	>16 (R)	>1 (R)	>4 (R)	>8 (R)	>1 (R)	>16 (R)	>4 (R)	>4/76 (R)	1 (S)	23 (S)
SAMPLE 27	>16 (R)	>1 (R)	>4 (R)	>8 (R)	>1 (R)	>16 (R)	>4 (R)	>4/76 (R)	1 (S)	20 (S)
SAMPLE 28	≤4 (S)	>1 (R)	>4 (R)	>8 (R)	>1 (R)	>16 (R)	>4 (R)	>4/76 (R)	1 (S)	23 (S)
SAMPLE 29	≤4 (S)	>1 (R)	>4 (R)	>8 (R)	>1 (R)	>16 (R)	>4 (R)	>4/76 (R)	2 (S)	15 (R)
SAMPLE 30	>16 (R)	>1 (R)	>4 (R)	>8 (R)	>1 (R)	>16 (R)	>4 (R)	>4/76 (R)	≤0.5 (S)	20 (S)
SAMPLE 31	>16 (R)	>1 (R)	>4 (R)	>8 (R)	>1 (R)	>16 (R)	>4 (R)	>4/76 (R)	≤0.5 (S)	21 (S)
SAMPLE 32	>16 (R)	>1 (R)	>4 (R)	>8 (R)	>1 (R)	>16 (R)	>4 (R)	>4/76 (R)	1 (S)	22 (S)
SAMPLE 33	>16 (R)	>1 (R)	>4 (R)	>8 (R)	>1 (R)	>16 (R)	>4 (R)	>4/76 (R)	1 (S)	23 (S)
SAMPLE 34	≤4 (S)	>1 (R)	>4 (R)	>8 (R)	>1 (R)	>16 (R)	>4 (R)	>4/76 (R)	0.5 (S)	23 (S)
SAMPLE 35	>16 (R)	>1 (R)	>4 (R)	>8 (R)	>1 (R)	>16 (R)	>4 (R)	>4/76 (R)	1 (S)	14 (R)
SAMPLE 36	>16 (R)	>1 (R)	>4 (R)	>8 (R)	>1 (R)	>16 (R)	>4 (R)	>4/76 (R)	1 (S)	21 (S)
SAMPLE 37	>16 (R)	>1 (R)	>4 (R)	>8 (R)	>1 (R)	>16 (R)	>4 (R)	>4/76 (R)	1 (S)	13 (R)
SAMPLE 38	>16 (R)	>1 (R)	>4 (R)	>8 (R)	>1 (R)	>16 (R)	>4 (R)	>4/76 (R)	1 (S)	21 (S)
SAMPLE 39	>16 (R)	>1 (R)	>4 (R)	>8 (R)	>1 (R)	>16 (R)	>4 (R)	>4/76 (R)	1 (S)	15 (R)
SAMPLE 40	>16 (R)	>1 (R)	>4 (R)	>8 (R)	>1 (R)	>16 (R)	>4 (R)	>4/76 (R)	0.5 (S)	23 (S)
SAMPLE 41	>16 (R)	>1 (R)	>4 (R)	>8 (R)	>1 (R)	>16 (R)	>4 (R)	>4/76 (R)	0.5 (S)	23 (S)
SAMPLE 42	>16 (R)	>1 (R)	>4 (R)	>8 (R)	>1 (R)	>16 (R)	>4 (R)	>4/76 (R)	1 (S)	20 (S)
SAMPLE 43	>16 (R)	>1 (R)	>4 (R)	>8 (R)	>1 (R)	>16 (R)	>4 (R)	>4/76 (R)	1 (S)	22 (S)
SAMPLE 44	>16 (R)	>1 (R)	>4 (R)	>8 (R)	>1 (R)	>16 (R)	>4 (R)	>4/76 (R)	1 (S)	21 (S)
SAMPLE 45	>16 (R)	>1 (R)	>4 (R)	>8 (R)	>1 (R)	>16 (R)	>4 (R)	>4/76 (R)	1 (S)	10 (R)
SAMPLE 46	>16 (R)	>1 (R)	>4 (R)	>8 (R)	>1 (R)	>16 (R)	>4 (R)	>4/76 (R)	2 (S)	20 (S)
SAMPLE 47	>16 (R)	>1 (R)	>4 (R)	>8 (R)	>1 (R)	>16 (R)	>4 (R)	>4/76 (R)	2 (S)	20 (S)
SAMPLE 48	>16 (R)	>1 (R)	>4 (R)	>8 (R)	>1 (R)	>16 (R)	>4 (R)	>4/76 (R)	1 (S)	22 (S)

S: susceptible; I: susceptible, increased exposure; R: resistant.

## Data Availability

Data are unavailable due to privacy and ethical restrictions.

## Supplementary Materials

The following supporting information can be downloaded at: https://www.mdpi.com/article/10.3390/microorganisms13112429/s1, Figure S1: Phylogenetic reconstruction of *Acinetobacter baumannii* strains including the reference genome of *Acinetobacter Baumanii* and SAMPLE-34; Figure S2: Phylogenetic representation of *Acinetobacter baumannii* strains which provides a visualisation of clusters even in the presence of SAMPLE-34. The tree branches highlighted in green have a support ≥0.9; Figure S3: Phylogenetic representation of *Acinetobacter baumannii* strains including *Acinetobacter baumannii* reference genome and excluding SAMPLE-34; Figure S4: Pangenome matrix of *Acinetobacter baumannii* strains which graphically shows the presence of highly specific genes in samples SAMPLE-34, SAMPLE-17 and SAMPLE-47. SAMPLE 16 also exhibited a gene pattern that differed marginally from its own cluster, resulting in slight resemblance to SAMPLE 34; Figure S5: The MDS graph illustration of the pangenome of the strains under analysis demonstrates the clear separation of the main clusters; Figure S6: Graph depicts the distribution of virulence factors as predicted by PathoFact; Table S1: Table containing information on the quality of contig and genome assembly, ST classification of strains, taxonomic classification, and description of genes specific to certain clusters and samples.; Table S2: The table contains statistical analysis to identify differences in the quantitative distribution of samples with respect to hospital department, site of infection and clusters. A table detailing each patient is also provided.

## Abbreviations

The following abbreviations are used in this manuscript:

**Abbreviation/Code**	**Definition**
aac(6′)-Ib′	aminoglycoside N-acetyltransferase AAC(6′)-Ib′
aadA1	ANT(3″)-Ia family aminoglycoside nucleotidyltransferase AadA1
abaF	fosfomycin efflux MFS transporter AbaF
adeC	multidrug efflux RND transporter AdeABC outer membrane channel subunit AdeC
AMR	antimicrobial resistance
amvA	multidrug efflux MFS transporter AmvA
ANI	average nucleotide identity
ant(3″)-IIa	aminoglycoside nucleotidyltransferase ANT(3″)-IIa
aph(3″)-Ib	aminoglycoside O-phosphotransferase APH(3″)-Ib
aph(3′)-Ia	aminoglycoside O-phosphotransferase APH(3′)-Ia
aph(6)-Id	aminoglycoside O-phosphotransferase APH(6)-Id
armA	16S rRNA (guanine(1405)-N(7))-methyltransferase ArmA
BAL	broncho-alveolar lavage
BfmRS	biofilm-controlling two-component regulatory system
blaADC-186	extended-spectrum class C beta-lactamase ADC-186
blaADC-30	extended-spectrum class C beta-lactamase ADC-30
blaADC-33	cefepime-hydrolyzing class C beta-lactamase ADC-33
blaADC-73	extended-spectrum class C beta-lactamase ADC-73
blaOXA-23	OXA-23 family carbapenem-hydrolyzing class D beta-lactamase OXA-23
blaOXA-66	OXA-51 family carbapenem-hydrolyzing class D beta-lactamase OXA-66
blaOXA-69	OXA-51 family carbapenem-hydrolyzing class D beta-lactamase OXA-69
blaOXA-82	OXA-51 family carbapenem-hydrolyzing class D beta-lactamase OXA-82
catB8	type B-3 chloramphenicol O-acetyltransferase CatB8
CheckM	tool for assessing genome quality (completeness/contamination)
ClusterPicker	software for automatic cluster identification in phylogenetic trees
Csu fimbriae	adhesion factor involved in biofilm formation and immune evasion
cxpE	chloramphenicol efflux transporter CxpE
Element symbol	element name
ftsI_A515V	Acinetobacter baumannii carbapenem/sulbactam-durlobactam resistant FtsI
GTDB-Tk	genome taxonomy database toolkit for taxonomic classification
gyrA_S81L	Acinetobacter baumannii quinolone resistant GyrA
ICU	intensive care unit
kSNP	tool for SNP-based phylogenetic analysis without a reference genome
MALDI-TOF	matrix-assisted laser desorption/ionization–time of flight mass spectrometry
MDR	multi-drug resistant
MEGAHIT	assembler for large and complex metagenomic datasets
MetaVF	tool using VFDB for virulence factor detection
MLST	multilocus sequence typing
mph(E)	Mph(E) family macrolide 2′-phosphotransferase
msr(E)	ABC-F type ribosomal protection protein Msr(E)
qacEdelta1	gene that confers resistance to disinfectants, specifically quaternary ammonium compounds.
ST	sequence type: identifier assigned to a specific allelic profile in the MLST scheme.
tetA	gene codifying for metal-tetracycline/H(+) antiporter conferring resistance to tetracycline by an active tetracycline efflux.
tetB	Tet(B) is a tetracycline efflux protein expressed in many Gram-negative bacteria. It confers resistance to tetracycline, doxycycline, and minocycline, but not tigecycline.

## References

[1] Asif M., Alvi I.A., Rehman S.U. (2018). Insight into *Acinetobacter baumannii*: Pathogenesis, Global Resistance, Mechanisms of Resistance, Treatment Options, and Alternative Modalities. Infect. Drug Resist..

[2] Wareth G., Linde J., Nguyen N.H., Nguyen T.N.M., Sprague L.D., Pletz M.W., Neubauer H. (2021). WGS-Based Analysis of Carbapenem-Resistant *Acinetobacter baumannii* in Vietnam and Molecular Characterization of Antimicrobial Determinants and MLST in Southeast Asia. Antibiotics.

[3] Tacconelli E., Carrara E., Savoldi A., Harbarth S., Mendelson M., Monnet D.L., Pulcini C., Kahlmeter G., Kluytmans J., Carmeli Y. (2018). Discovery, Research, and Development of New Antibiotics: The WHO Priority List of Antibiotic-Resistant Bacteria and Tuberculosis. Lancet Infect. Dis..

[4] Diancourt L., Passet V., Nemec A., Dijkshoorn L., Brisse S. (2010). The Population Structure of *Acinetobacter baumannii*: Expanding Multiresistant Clones from an Ancestral Susceptible Genetic Pool. PLoS ONE.

[5] Mellace M., Ceniti C., Paonessa M., Procopio A.C., Tilocca B. (2024). Multidrug-Resistant *Acinetobacter baumannii*: An Underestimated Pathogen in Veterinary Medicine in Italy. Ger. J. Vet. Res..

[6] Santajit S., Indrawattana N. (2016). Mechanisms of Antimicrobial Resistance in ESKAPE Pathogens. Biomed. Res. Int..

[7] Hujer K.M., Hujer A.M., Hulten E.A., Bajaksouzian S., Adams J.M., Donskey C.J., Ecker D.J., Massire C., Eshoo M.W., Sampath R. (2006). Analysis of Antibiotic Resistance Genes in Multidrug-Resistant *Acinetobacter* sp. Isolates from Military and Civilian Patients Treated at the Walter Reed Army Medical Center. Antimicrob. Agents Chemother..

[8] Nemec A., Dijkshoorn L., Van Der Reijden T.J.K. (2004). Long-Term Predominance of Two Pan-European Clones among Multi-Resistant *Acinetobacter baumannii* Strains in the Czech Republic. J. Med. Microbiol..

[9] Kyriakidis I., Vasileiou E., Pana Z.D., Tragiannidis A. (2021). *Acinetobacter baumannii* Antibiotic Resistance Mechanisms. Pathogens.

[10] Furuya E.Y., Lowy F.D. (2006). Antimicrobial-Resistant Bacteria in the Community Setting. Nat. Rev. Microbiol..

[11] Cave R., Cole J., Mkrtchyan H.V. (2021). Surveillance and Prevalence of Antimicrobial Resistant Bacteria from Public Settings within Urban Built Environments: Challenges and Opportunities for Hygiene and Infection Control. Environ. Int..

[12] Almasaudi S.B. (2018). *Acinetobacter* spp. as Nosocomial Pathogens: Epidemiology and Resistance Features. Saudi J. Biol. Sci..

[13] Peacock S.J., Parkhill J., Brown N.M. (2018). Changing the Paradigm for Hospital Outbreak Detection by Leading with Genomic Surveillance of Nosocomial Pathogens. Microbiology.

[14] Aldeyab M.A., Harbarth S., Vernaz N., Kearney M.P., Scott M.G., Darwish Elhajji F.W., Aldiab M.A., McElnay J.C. (2012). The Impact of Antibiotic Use on the Incidence and Resistance Pattern of Extended-spectrum Beta-lactamase-producing Bacteria in Primary and Secondary Healthcare Settings. Br. J. Clin. Pharma..

[15] Karanika S., Karantanos T., Arvanitis M., Grigoras C., Mylonakis E. (2016). Fecal Colonization with Extended-Spectrum Beta-Lactamase–Producing *Enterobacteriaceae* and Risk Factors Among Healthy Individuals: A Systematic Review and Metaanalysis. Clin. Infect. Dis..

[16] Zeana C., Larson E., Sahni J., Bayuga S.J., Wu F., Della-Latta P. (2003). The Epidemiology of Multidrug-Resistant *Acinetobacter baumannii* Does the Community Represent a Reservoir?. Infect. Control Hosp. Epidemiol..

[17] Ciccozzi M., Cella E., Lai A., Florio L., Antonelli F., Fogolari M., Di Matteo F.M., Pizzicannella M., Colombo B., Dicuonzo G. (2019). Phylogenetic Analysis of Multi-Drug Resistant *Klebsiella pneumoniae* Strains from Duodenoscope Biofilm: Microbiological Surveillance and Reprocessing Improvements for Infection Prevention. Front. Public Health.

[18] Angeletti S., Cella E., Lai A., Lo Presti A., Antonelli F., Conti A., Lopalco M., Spoto S., Zehender G., Ciccozzi M. (2017). Whole-Genome Sequencing of *Klebsiella pneumoniae* MDR Strain Isolated in a Syrian Refugee. Pathog. Glob. Health.

[19] Harding C.M., Hennon S.W., Feldman M.F. (2018). Uncovering the Mechanisms of *Acinetobacter baumannii* Virulence. Nat. Rev. Microbiol..

[20] Ristori M.V., Scarpa F., Sanna D., Casu M., Petrosillo N., Longo U.G., Lucia D.F., Spoto S., Chiantia R.M., Caserta A. (2024). Multidrug-Resistant *Klebsiella pneumoniae* Strains in a Hospital: Phylogenetic Analysis to Investigate Local Epidemiology. Microorganisms.

[21] Angeletti S., Dicuonzo G., Lo Presti A., Cella E., Crea F., Avola A., Vitali M.A., Fagioni M., De Florio L. (2015). MALDI-TOF Mass Spectrometry and Blakpc Gene Phylogenetic Analysis of an Outbreak of Carbapenem-Resistant *K. pneumoniae* Strains. New Microbiol..

[22] (2019). Susceptibility testing of infectious agents and evaluation of performance of antimicrobial susceptibility test devicesPart 1: Broth micro-dilution reference method for testing the in vitro activity of antimicrobial agents against rapidly growing aerobic bacteria involved in infectious diseases.

[23] Sserwadda I., Mboowa G. (2021). rMAP: The Rapid Microbial Analysis Pipeline for ESKAPE Bacterial Group Whole-Genome Sequence Data. Microb. Genom..

[24] Bolger A.M., Lohse M., Usadel B. (2014). Trimmomatic: A Flexible Trimmer for Illumina Sequence Data. Bioinformatics.

[25] Li D., Liu C.-M., Luo R., Sadakane K., Lam T.-W. (2015). MEGAHIT: An Ultra-Fast Single-Node Solution for Large and Complex Metagenomics Assembly via Succinct *de Bruijn* Graph. Bioinformatics.

[26] Alneberg J., Bjarnason B.S., De Bruijn I., Schirmer M., Quick J., Ijaz U.Z., Lahti L., Loman N.J., Andersson A.F., Quince C. (2014). Binning Metagenomic Contigs by Coverage and Composition. Nat. Methods.

[27] Mikheenko A., Prjibelski A., Saveliev V., Antipov D., Gurevich A. (2018). Versatile Genome Assembly Evaluation with QUAST-LG. Bioinformatics.

[28] Chaumeil P.-A., Mussig A.J., Hugenholtz P., Parks D.H. (2022). GTDB-Tk v2: Memory Friendly Classification with the Genome Taxonomy Database. Bioinformatics.

[29] Figueras M.J., Beaz-Hidalgo R., Hossain M.J., Liles M.R. (2014). Taxonomic Affiliation of New Genomes Should Be Verified Using Average Nucleotide Identity and Multilocus Phylogenetic Analysis. Genome Announc..

[30] Bowers R.M., Kyrpides N.C., Stepanauskas R., Harmon-Smith M., Doud D., Reddy T.B.K., Schulz F., Jarett J., Rivers A.R., Eloe-Fadrosh E.A. (2017). Minimum information about a single amplified genome (MISAG) and a metagenome-assembled genome (MIMAG) of bacteria and archaea. Nat. Biotechnol..

[31] Parks D.H., Imelfort M., Skennerton C.T., Hugenholtz P., Tyson G.W. (2015). CheckM: Assessing the Quality of Microbial Genomes Recovered from Isolates, Single Cells, and Metagenomes. Genome Res..

[32] Prodhan A. Asadprodhan/Average-Nucleotide-Identity-ANI-Analysis 2025. https://github.com/asadprodhan/Average-Nucleotide-Identity-ANI-analysis.

[33] Seemann T. Tseemann/Mlst 2025. https://github.com/tseemann/mlst.

[34] Shakya M., Ahmed S.A., Davenport K.W., Flynn M.C., Lo C.-C., Chain P.S.G. (2020). Standardized Phylogenetic and Molecular Evolutionary Analysis Applied to Species across the Microbial Tree of Life. Sci. Rep..

[35] Hall B.G., Nisbet J. (2023). Building Phylogenetic Trees from Genome Sequences With kSNP4. Mol. Biol. Evol..

[36] Ragonnet-Cronin M., Hodcroft E., Hué S., Fearnhill E., Delpech V., Brown A.J.L., Lycett S. (2013). Automated Analysis of Phylogenetic Clusters. BMC Bioinform..

[37] FigTree. http://tree.bio.ed.ac.uk/software/figtree/.

[38] Page A.J., Cummins C.A., Hunt M., Wong V.K., Reuter S., Holden M.T.G., Fookes M., Falush D., Keane J.A., Parkhill J. (2015). Roary: Rapid Large-Scale Prokaryote Pan Genome Analysis. Bioinformatics.

[39] Afgan E., Baker D., Batut B., van den Beek M., Bouvier D., Čech M., Chilton J., Clements D., Coraor N., Grüning B.A. (2018). The Galaxy Platform for Accessible, Reproducible and Collaborative Biomedical Analyses: 2018 Update. Nucleic Acids Res..

[40] Roary/Contrib/Roary_plots/Roary_plots.Py at Master. https://github.com/sanger-pathogens/Roary/blob/master/contrib/roary_plots/roary_plots.py.

[41] Powell D. Drpowell/FriPan 2024.

[42] Feldgarden M., Brover V., Gonzalez-Escalona N., Frye J.G., Haendiges J., Haft D.H., Hoffmann M., Pettengill J.B., Prasad A.B., Tillman G.E. (2021). AMRFinderPlus and the Reference Gene Catalog Facilitate Examination of the Genomic Links among Antimicrobial Resistance, Stress Response, and Virulence. Sci. Rep..

[43] Seemann T. (2014). Prokka: Rapid Prokaryotic Genome Annotation. Bioinformatics.

[44] De Nies L., Lopes S., Busi S.B., Galata V., Heintz-Buschart A., Laczny C.C., May P., Wilmes P. (2021). PathoFact: A Pipeline for the Prediction of Virulence Factors and Antimicrobial Resistance Genes in Metagenomic Data. Microbiome.

[45] Dong W., Fan X., Guo Y., Wang S., Jia S., Lv N., Yuan T., Pan Y., Xue Y., Chen X. (2024). An Expanded Database and Analytical Toolkit for Identifying Bacterial Virulence Factors and Their Associations with Chronic Diseases. Nat. Commun..

[46] Gu Z. (2022). Complex Heatmap Visualization. iMeta.

[47] Posit The Data Science Code Editor. https://www.posit.co/.

[48] Patil I. (2021). Visualizations with Statistical Details: The “ggstatsplot” Approach. J. Open Source Softw..

[49] Xu S., Dai Z., Guo P., Fu X., Liu S., Zhou L., Tang W., Feng T., Chen M., Zhan L. (2021). ggtreeExtra: Compact Visualization of Richly Annotated Phylogenetic Data. Mol. Biol. Evol..

[50] Yu G. (2022). Data Integration, Manipulation and Visualization of Phylogenetic Trees.

[51] Wareth G., Brandt C., Sprague L.D., Neubauer H., Pletz M.W. (2020). Spatio-Temporal Distribution of *Acinetobacter baumannii* in Germany—A Comprehensive Systematic Review of Studies on Resistance Development in Humans (2000–2018). Microorganisms.

[52] Wareth G., Abdel-Glil M.Y., Schmoock G., Steinacker U., Kaspar H., Neubauer H., Sprague L.D. (2019). Draft Genome Sequence of an *Acinetobacter baumannii* Isolate Recovered from a Horse with Conjunctivitis in Germany. Microbiol. Resour. Announc..

[53] Wareth G., Linde J., Hammer P., Nguyen N.H., Nguyen T.N.M., Splettstoesser W.D., Makarewicz O., Neubauer H., Sprague L.D., Pletz M.W. (2020). Phenotypic and WGS-Derived Antimicrobial Resistance Profiles of Clinical and Non-Clinical *Acinetobacter baumannii* Isolates from Germany and Vietnam. Int. J. Antimicrob. Agents.

[54] Wareth G., Neubauer H., Sprague L.D. (2019). *Acinetobacter baumannii*–a Neglected Pathogen in Veterinary and Environmental Health in Germany. Vet. Res. Commun..

[55] Murugaiyan J., Walther B., Stamm I., Abou-Elnaga Y., Brueggemann-Schwarze S., Vincze S., Wieler L.H., Lübke-Becker A., Semmler T., Roesler U. (2014). Species Differentiation within the Staphylococcus Intermedius Group Using a Refined MALDI-TOF MS Database. Clin. Microbiol. Infect..

[56] Vijayakumar S., Biswas I., Veeraraghavan B. (2019). Accurate Identification of Clinically Important *Acinetobacter* spp.: An Update. Future Sci. OA.

[57] Wendel A.F., Malecki M., Otchwemah R., Tellez-Castillo C.J., Sakka S.G., Mattner F. (2018). One-Year Molecular Surveillance of Carbapenem-Susceptible *A. Baumannii* on a German Intensive Care Unit: Diversity or Clonality. Antimicrob. Resist. Infect. Control..

[58] Higgins P.G., Schneiders T., Hamprecht A., Seifert H. (2010). In Vivo Selection of a Missense Mutation in adeR and Conversion of the Novel *Bla*_OXA-164_ Gene into *Bla*_OXA-58_ in Carbapenem-Resistant *Acinetobacter baumannii* Isolates from a Hospitalized Patient. Antimicrob. Agents Chemother..

[59] Corbella X., Pujol M., Ayats J., Sendra M., Ardanuy C., Dominguez M.A., Linares J., Ariza J., Gudiol F. (1996). Relevance of Digestive Tract Colonization in the Epidemiology of Nosocomial Infections Due to Multiresistant *Acinetobacter baumannii*. Clin. Infect. Dis..

[60] Ayats J., Corbella X., Ardanuy C., Domínguez M.A., Ricart A., Ariza J., Martin R., Liñares J. (1997). Epidemiological Significance of Cutaneous, Pharyngeal, and Digestive Tract Colonization by Multiresistant *Acinetobacter baumannii* in ICU Patients. J. Hosp. Infect..

[61] Odih E.E., Irek E.O., Obadare T.O., Oaikhena A.O., Afolayan A.O., Underwood A., Adenekan A.T., Ogunleye V.O., Argimon S., Dalsgaard A. (2022). Rectal Colonization and Nosocomial Transmission of Carbapenem-Resistant *Acinetobacter baumannii* in an Intensive Care Unit, Southwest Nigeria. Front. Med..

[62] Larsen M.V., Cosentino S., Rasmussen S., Friis C., Hasman H., Marvig R.L., Jelsbak L., Sicheritz-Pontén T., Ussery D.W., Aarestrup F.M. (2012). Multilocus Sequence Typing of Total-Genome-Sequenced Bacteria. J. Clin. Microbiol..

[63] Wareth G., Linde J., Hammer P., Splettstoesser W.D., Pletz M.W., Neubauer H., Sprague L.D. (2021). Molecular Characterization of German *Acinetobacter baumannii* Isolates and Multilocus Sequence Typing (MLST) Analysis Based on WGS Reveals Novel STs. Pathogens.

[64] Hamidian M., Nigro S.J. (2019). Emergence, Molecular Mechanisms and Global Spread of Carbapenem-Resistant *Acinetobacter baumannii*. Microb. Genom..

[65] Reis A.C., Cunha M.V. (2021). The Open Pan-Genome Architecture and Virulence Landscape of *Mycobacterium Bovis*. Microb. Genom..

[66] Medini D., Donati C., Tettelin H., Masignani V., Rappuoli R. (2005). The Microbial Pan-Genome. Curr. Opin. Genet. Dev..

[67] Vernikos G., Medini D., Riley D.R., Tettelin H. (2015). Ten Years of Pan-Genome Analyses. Curr. Opin. Microbiol..

[68] Chan A.P., Sutton G., DePew J., Krishnakumar R., Choi Y., Huang X.-Z., Beck E., Harkins D.M., Kim M., Lesho E.P. (2015). A Novel Method of Consensus Pan-Chromosome Assembly and Large-Scale Comparative Analysis Reveal the Highly Flexible Pan-Genome of *Acinetobacter baumannii*. Genome Biol..

[69] O’Brien B., Yushchenko A., Suh J., Jung D., Cai Z., Nguyen N.S., Semret M., Dufour S., Fanning S., Ronholm J. (2025). Subtle Genomic Differences in *Klebsiella pneumoniae* Sensu Stricto Isolates Indicate Host Adaptation. One Health.

[70] Karampatakis T., Tsergouli K., Behzadi P. (2024). Pan-Genome Plasticity and Virulence Factors: A Natural Treasure Trove for *Acinetobacter baumannii*. Antibiotics.

[71] Marino A., Augello E., Stracquadanio S., Bellanca C.M., Cosentino F., Spampinato S., Cantarella G., Bernardini R., Stefani S., Cacopardo B. (2024). Unveiling the Secrets of *Acinetobacter baumannii*: Resistance, Current Treatments, and Future Innovations. Int. J. Mol. Sci..

[72] Zack K.M., Sorenson T., Joshi S.G. (2024). Types and Mechanisms of Efflux Pump Systems and the Potential of Efflux Pump Inhibitors in the Restoration of Antimicrobial Susceptibility, with a Special Reference to *Acinetobacter baumannii*. Pathogens.

[73] Ahmad I., Nadeem A., Mushtaq F., Zlatkov N., Shahzad M., Zavialov A.V., Wai S.N., Uhlin B.E. (2023). Csu Pili Dependent Biofilm Formation and Virulence of *Acinetobacter baumannii*. NPJ Biofilms Microbiomes.

[74] Kim H.-J., Kim N.-Y., Ko S.-Y., Park S.-Y., Oh M.-H., Shin M.-S., Lee Y.-C., Lee J.-C. (2022). Complementary Regulation of BfmRS Two-Component and AbaIR Quorum Sensing Systems to Express Virulence-Associated Genes in *Acinetobacter baumannii*. Int. J. Mol. Sci..

[75] Morris F.C., Dexter C., Kostoulias X., Uddin M.I., Peleg A.Y. (2019). The Mechanisms of Disease Caused by *Acinetobacter baumannii*. Front. Microbiol..

[76] Fiester S.E., Arivett B.A., Schmidt R.E., Beckett A.C., Ticak T., Carrier M.V., Ghosh R., Ohneck E.J., Metz M.L., Sellin Jeffries M.K. (2016). Iron-Regulated Phospholipase C Activity Contributes to the Cytolytic Activity and Virulence of *Acinetobacter baumannii*. PLoS ONE.

