# Supplementary material for: Genomic Surveillance and Resistance Profiling of Multidrug-Resistant Acinetobacter baumannii Clinical Isolates: Clonal Diversity and Virulence Insights
Source: Microorganisms. 2025 Oct 23;13(11):2429. doi: 10.3390/microorganisms13112429 (PMC12654848; doi:10.3390/microorganisms13112429)
Supplement: Supplementary file 1 [file microorganisms-13-02429-s001.zip › Supplementary_File_Acinetobacter.pdf]

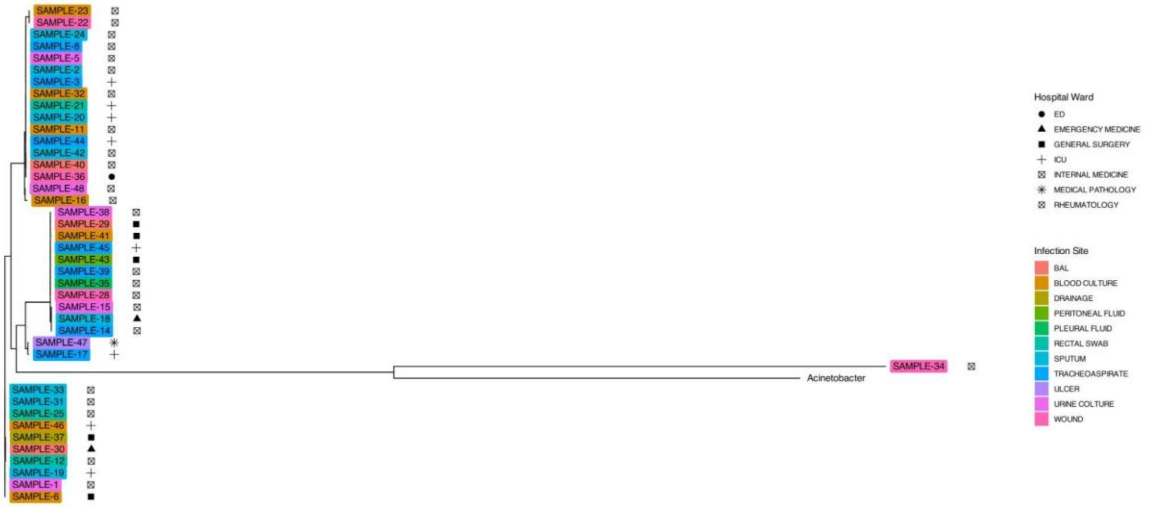

Figure S1. Phylogenetic reconstruction of *Acinetobacter baumannii* strains including the reference genome of *Acinetobacter Baumanii* and SAMPLE-34.

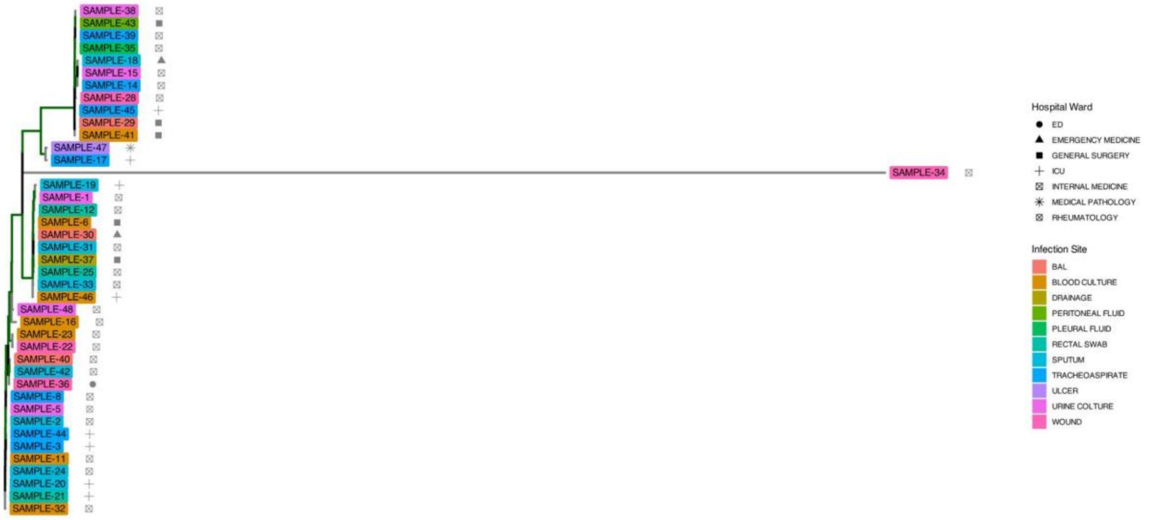

Figure S2. Phylogenetic representation of *Acinetobacter baumannii* strains which provides a visualisation of clusters even in the presence of SAMPLE-34. The tree branches highlighted in green have a support ≥ 0.9.

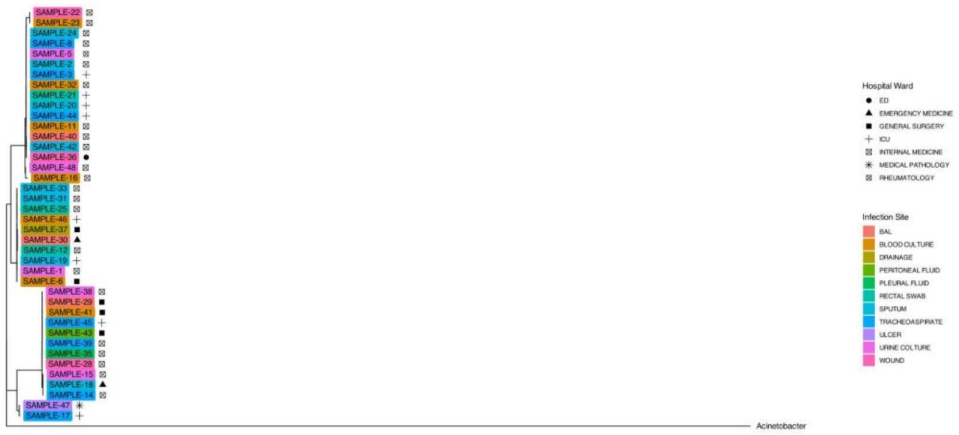

Figure S3. Phylogenetic representation of *Acinetobacter baumannii* strains including *Acinetobacter baumannii* reference genome and excluding SAMPLE-34.

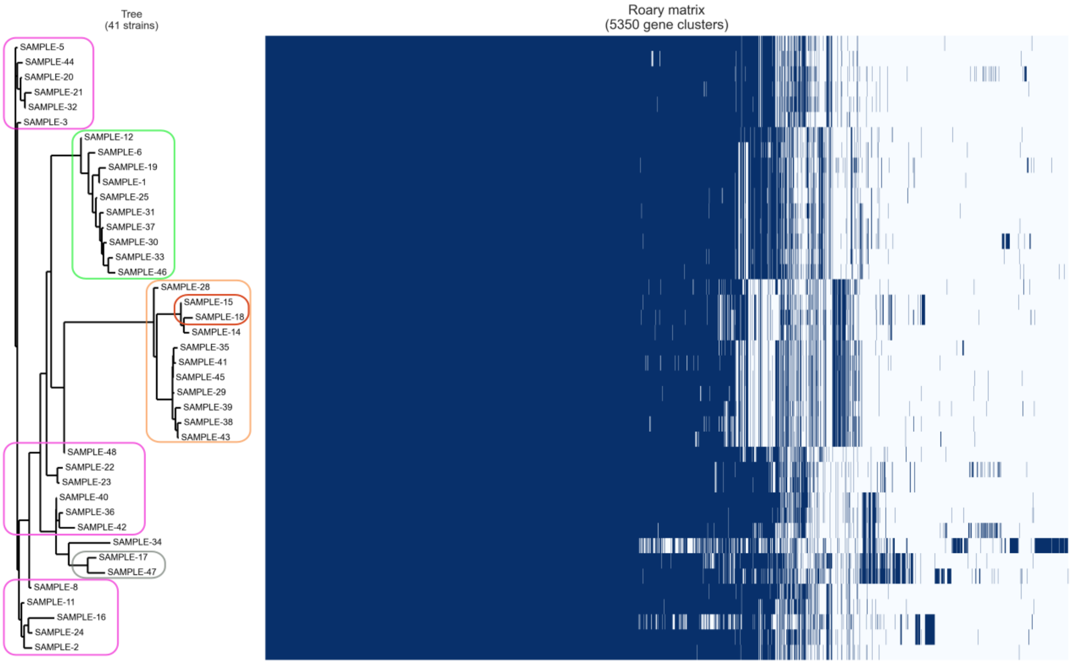

Figure S4. Pangenome matrix of *Acinetobacter baumannii* strains which graphically shows the presence of highly specific genes in samples SAMPLE-34, SAMPLE-17 and SAMPLE-47. SAMPLE 16 also exhibited a gene pattern that differed marginally from its own cluster, resulting in slight resemblance to SAMPLE 34.

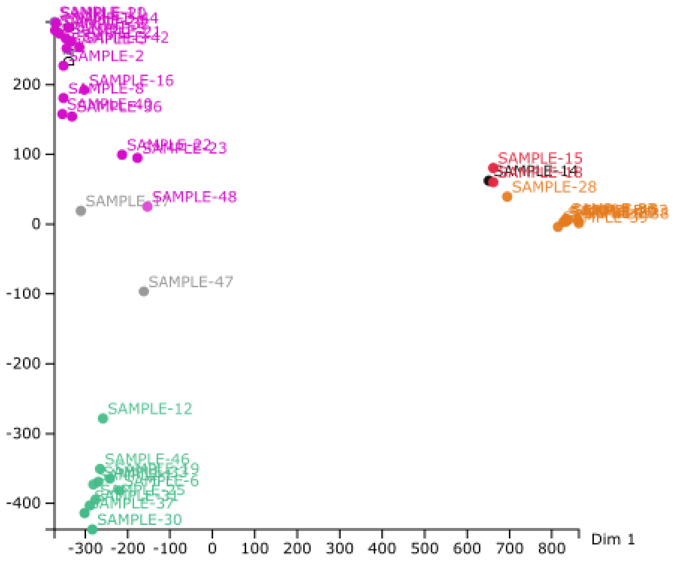

Figure S5. The MDS graph illustration of the pangenome of the strains under analysis demonstrates the clear separation of the main clusters.

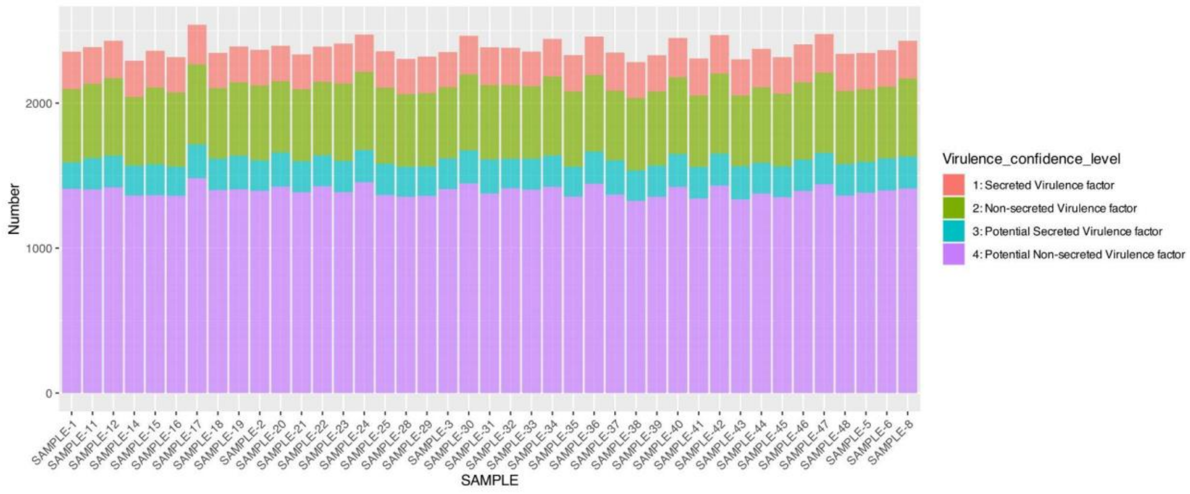

Figure S6. Graph depicts the distribution of virulence factors as predicted by PathoFact.
